# 1-[6-(1*H*-Indol-1-yl)pyridin-2-yl]-1*H*-indole-3-carbaldehyde

**DOI:** 10.1107/S1600536813034375

**Published:** 2014-01-04

**Authors:** C. Ramathilagam, P. R. Umarani, N. Venkatesan, P. Rajakumar, B. Gunasekaran, V. Manivannan

**Affiliations:** a Department of Physics, AMET University, Kanathur, Chennai 603 112, India; b Principal, Kundavai Nachiyar Govt College for Women, Thanjavur 613 007, India; c Department of Organic Chemistry, University of Madras, Guindy campus, Chennai 600 025, India; d Department of Physics & Nano Technology, SRM University, SRM Nagar, Kattankulathur, Kancheepuram Dist, Chennai 603 203, Tamil Nadu, India; e Department of Research and Development, PRIST University, Vallam, Thanjavur 613 403, Tamil Nadu, India

## Abstract

In the title compound, C_22_H_15_N_3_O, the dihedral angle between the two indole units is 33.72 (3)°. The mol­ecular structure features a weak intra­molecular C—H⋯N inter­action. In the crystal, weak C—H⋯O and C—H⋯π inter­actions, forming a two-dimensional network parallel to the *bc* plane.

## Related literature   

For the biological activity of indole derivatives, see: Macor *et al.* (1992 ▶); Andreani *et al.* (2001 ▶); Quetin-Leclercq (1994 ▶); Mukhopadhyay *et al.* (1981 ▶); Singh *et al.* (2000 ▶). For related structures see: Dileep *et al.* (2012 ▶); Wu *et al.* (2012 ▶)
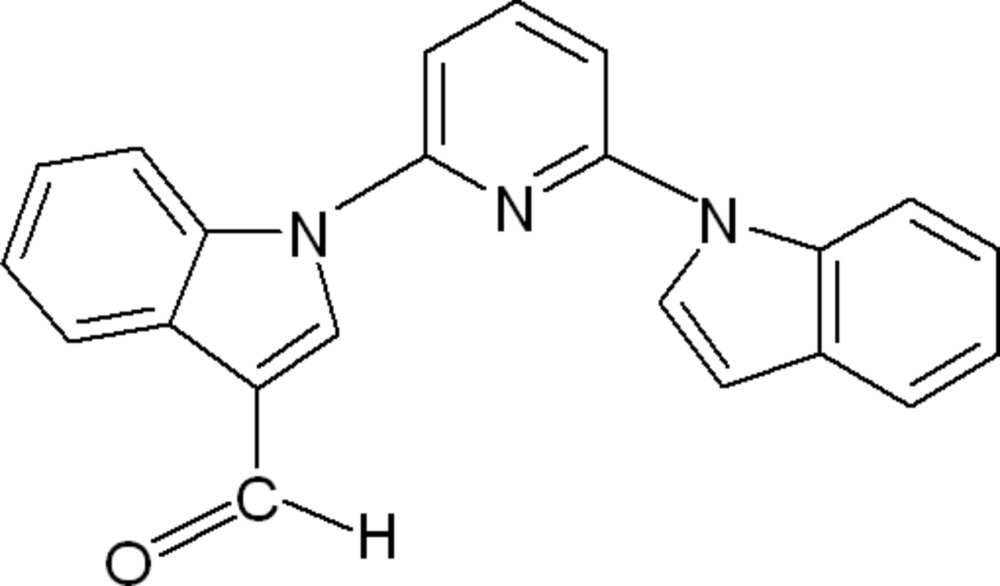


## Experimental   

### 

#### Crystal data  

C_22_H_15_N_3_O
*M* *_r_* = 337.37
Orthorhombic, 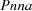

*a* = 18.2208 (7) Å
*b* = 15.7672 (9) Å
*c* = 11.7034 (7) Å
*V* = 3362.3 (3) Å^3^
*Z* = 8
Mo *K*α radiation
μ = 0.08 mm^−1^
*T* = 295 K
0.25 × 0.20 × 0.15 mm

#### Data collection  

Bruker APEXII CCD diffractometer
Absorption correction: multi-scan (*SADABS*; Sheldrick, 1996 ▶) *T*_min_ = 0.980, *T*_max_ = 0.987
11189 measured reflections
3521 independent reflections
2072 reflections with *I* > 2σ(*I*)
*R*_int_ = 0.031

#### Refinement  

*R*[*F* ^2^ > 2σ(*F* ^2^)] = 0.046
*wR*(*F* ^2^) = 0.118
*S* = 1.00
3521 reflections
235 parameters
H-atom parameters constrained
Δρ_max_ = 0.15 e Å^−3^
Δρ_min_ = −0.16 e Å^−3^

### 

Data collection: *APEX2* (Bruker, 2008 ▶); cell refinement: *SAINT* (Bruker, 2008 ▶); data reduction: *SAINT*; program(s) used to solve structure: *SHELXS97* (Sheldrick, 2008 ▶); program(s) used to refine structure: *SHELXL97* (Sheldrick, 2008 ▶); molecular graphics: *PLATON* (Spek, 2009 ▶); software used to prepare material for publication: *SHELXL97*.

## Supplementary Material

Crystal structure: contains datablock(s) I. DOI: 10.1107/S1600536813034375/hg5369sup1.cif

Structure factors: contains datablock(s) I. DOI: 10.1107/S1600536813034375/hg5369Isup2.hkl

Supporting information file. DOI: 10.1107/S1600536813034375/hg5369Isup3.cml

CCDC reference:

Additional supporting information:  crystallographic information; 3D view; checkCIF report

## Figures and Tables

**Table 1:** Hydrogen-bond geometry (Å, °) *Cg*5 is the centroid of the C16–C21 ring.

*D*—H⋯*A*	*D*—H	H⋯*A*	*D*⋯*A*	*D*—H⋯*A*
C6—H6⋯O1^i^	0.93	2.50	3.227 (3)	135
C12—H12⋯N1	0.93	2.41	2.931 (2)	116
C2—H2⋯*Cg*5^ii^	0.93	2.73	3.554 (2)	148

Symmetry codes: (i) 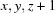; (ii) 
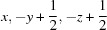
.

**Fig. 1.**
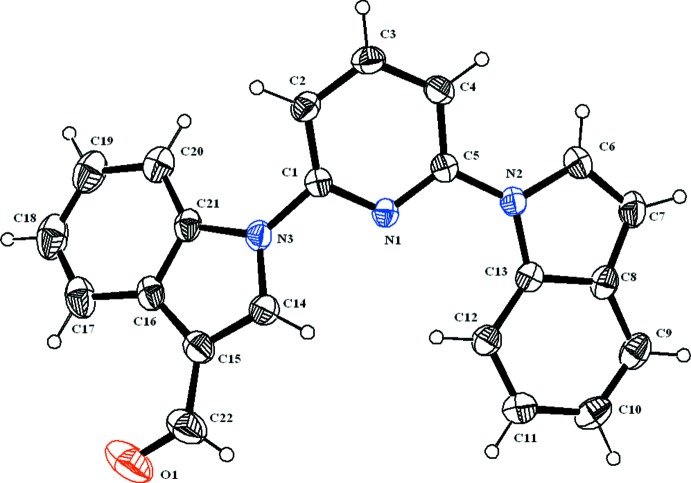
The molecular structure of (I), with atom labels and 30% probability displacement ellipsoids for non-H atoms.

**Fig. 2.**
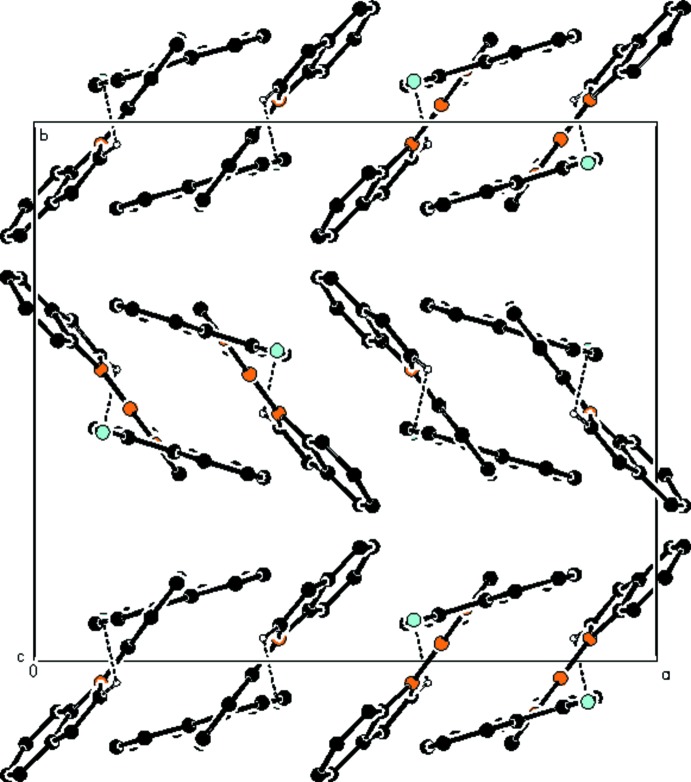
The packing of (I), viewed down the c axis. Hydrogen bonds are shown as dashed lines.

## supplementary crystallographic information

### 1. Comment

The chemistry of indole has been of increasing interest, since several compounds of this type possess diverse biological activities (Macor *et al.*, 1992). These derivatives exhibit antibacterial, antifungal (Singh *et al.*, 2000) and antitumour activities (Andreani *et al.*, 2001). Some of the indole alkaloids extracted from plants possess interesting cytotoxic and antiparasitic properties (Quetin-Leclercq, 1994; Mukhopadhyay *et al.*, 1981).

The geometric parameters of the title molecule (Fig. 1) agree well with reported similar structure (Dileep *et al.*, 2012; Wu *et al.*, 2012). The dihedral angle between the two indole moieties is 33.72 (3) °. The molecular structure is stabilized by weak intramolecular C—H···N interaction and the crystal packing is controlled by weak intermolecular C—H···O and C—H···π [C2—H2···*Cg*5(*x*, 1/2 - *y*, 1/2 - *z*) distance of 3.554 (2) Å, (*Cg*5 is the centroid of the ring defined by the atoms C16—C21)] interactions.

### 2. Experimental

To a stirred solution of dimethylformamide (2.10g, 28.70 mmol) at 0°C, added phosphorous oxycholoride (1.10g, 7.17 mmol) drop wise under nitrogen atmosphere. 2,6-bis(*N*-indolyl)pyridine (1.0g, 3.23 mmol) in dimethylformamide was then added to the reaction at 0°C to 10°C. After the completion of addition, the reaction mixture was allow to attain room temperature and then stirred for additional one hour at 35°C. The reaction was then quenched by adding crushed ice (100 g) and further water (100 ml). Then the reaction mixture was then treated thrice with NaOH solution (1*M*). The reaction mixture was heated after adding one portion of NaOH solution and the rest of the two portions were added later with stirring. The reaction mixture was then kept in refrigerator overnight. The precipitate obtained was collected by filtration and then dissolved in chloroform (2 *x* 100 ml). The organic layer was then dried over (Na_2_SO_4_), filtered and solvent was evaporated under reduced pressure to give the residue which was then chromatographed over SiO_2_ using hexane: chloroform (1:4) as eluting solvent to give the mono aldehyde(27%) as a colourless solid and further elution with CHCl_3_: methanol (99:1) afforded the corresponding dialdehyde (55%).

### 3. Refinement

H atoms were positioned geometrically and refined using riding model with C—H = 0.93 Å and *U*_iso_(H) = 1.2Ueq(C) for aromatic C—H.

### Crystal data

**Table:**

C_22_H_15_N_3_O	*F*(000) = 1408
*M**_r_* = 337.37	*D*_x_ = 1.333 Mg m^−^^3^
Orthorhombic, *P**n**n**a*	Mo *K*α radiation, λ = 0.71073 Å
Hall symbol: -P 2a 2bc	Cell parameters from 3755 reflections
*a* = 18.2208 (7) Å	θ = 2.0–26.6°
*b* = 15.7672 (9) Å	µ = 0.08 mm^−^^1^
*c* = 11.7034 (7) Å	*T* = 295 K
*V* = 3362.3 (3) Å^3^	Block, colourless
*Z* = 8	0.25 × 0.20 × 0.15 mm

### Data collection

**Table:**

Bruker APEXII CCD diffractometer	3521 independent reflections
Radiation source: fine-focus sealed tube	2072 reflections with *I* > 2σ(*I*)
Graphite monochromator	*R*_int_ = 0.031
Detector resolution: 0 pixels mm^-1^	θ_max_ = 26.7°, θ_min_ = 2.1°
ω and φ scans	*h* = −14→22
Absorption correction: multi-scan (*SADABS*; Sheldrick, 1996)	*k* = −19→13
*T*_min_ = 0.980, *T*_max_ = 0.987	*l* = −14→10
11189 measured reflections	

### Refinement

**Table:**

Refinement on *F*^2^	Primary atom site location: structure-invariant direct methods
Least-squares matrix: full	Secondary atom site location: difference Fourier map
*R*[*F*^2^ > 2σ(*F*^2^)] = 0.046	Hydrogen site location: inferred from neighbouring sites
*wR*(*F*^2^) = 0.118	H-atom parameters constrained
*S* = 1.00	*w* = 1/[σ^2^(*F*_o_^2^) + (0.0507*P*)^2^ + 0.3404*P*] where *P* = (*F*_o_^2^ + 2*F*_c_^2^)/3
3521 reflections	(Δ/σ)_max_ < 0.001
235 parameters	Δρ_max_ = 0.15 e Å^−^^3^
0 restraints	Δρ_min_ = −0.16 e Å^−^^3^

### Fractional atomic coordinates and isotropic or equivalent isotropic displacement parameters (Å^2^)

**Table:**

	*x*	*y*	*z*	*U*_iso_*/*U*_eq_	
C1	0.19452 (8)	0.09535 (11)	0.38032 (14)	0.0407 (4)	
C2	0.23265 (9)	0.15297 (11)	0.44461 (16)	0.0493 (5)	
H2	0.2599	0.1961	0.4109	0.059*	
C3	0.22882 (10)	0.14407 (12)	0.56135 (16)	0.0541 (5)	
H3	0.2545	0.1812	0.6083	0.065*	
C4	0.18729 (10)	0.08067 (12)	0.60890 (15)	0.0506 (5)	
H4	0.1840	0.0745	0.6877	0.061*	
C5	0.15023 (8)	0.02580 (10)	0.53543 (14)	0.0408 (4)	
C6	0.10521 (10)	−0.06632 (14)	0.69106 (16)	0.0595 (5)	
H6	0.1322	−0.0413	0.7495	0.071*	
C7	0.05930 (11)	−0.13186 (14)	0.70475 (18)	0.0658 (6)	
H7	0.0495	−0.1599	0.7730	0.079*	
C8	0.02821 (9)	−0.15088 (12)	0.59656 (16)	0.0510 (5)	
C9	−0.02337 (10)	−0.21026 (13)	0.55946 (19)	0.0619 (6)	
H9	−0.0439	−0.2484	0.6109	0.074*	
C10	−0.04334 (10)	−0.21191 (13)	0.4474 (2)	0.0639 (6)	
H10	−0.0771	−0.2520	0.4222	0.077*	
C11	−0.01383 (10)	−0.15451 (13)	0.37027 (18)	0.0598 (5)	
H11	−0.0286	−0.1565	0.2943	0.072*	
C12	0.03711 (9)	−0.09435 (12)	0.40377 (16)	0.0500 (5)	
H12	0.0564	−0.0557	0.3518	0.060*	
C13	0.05850 (9)	−0.09360 (10)	0.51763 (15)	0.0417 (4)	
C14	0.13419 (11)	0.07765 (11)	0.19300 (16)	0.0528 (5)	
H14	0.0891	0.0604	0.2220	0.063*	
C15	0.15005 (12)	0.08533 (11)	0.07958 (16)	0.0579 (5)	
C16	0.22504 (11)	0.11196 (11)	0.07240 (16)	0.0530 (5)	
C17	0.27264 (15)	0.12907 (13)	−0.01922 (19)	0.0745 (7)	
H17	0.2566	0.1241	−0.0943	0.089*	
C18	0.34277 (16)	0.15310 (15)	0.0044 (2)	0.0852 (8)	
H18	0.3745	0.1651	−0.0557	0.102*	
C19	0.36810 (13)	0.16008 (14)	0.1154 (2)	0.0807 (7)	
H19	0.4164	0.1765	0.1283	0.097*	
C20	0.32270 (11)	0.14309 (12)	0.20799 (19)	0.0610 (5)	
H20	0.3396	0.1470	0.2828	0.073*	
C21	0.25168 (10)	0.12027 (10)	0.18389 (15)	0.0489 (5)	
C22	0.09838 (16)	0.06891 (14)	−0.0091 (2)	0.0804 (7)	
H22	0.0518	0.0513	0.0129	0.096*	
N1	0.15424 (7)	0.03227 (8)	0.42256 (11)	0.0408 (4)	
N2	0.10689 (7)	−0.04099 (9)	0.57769 (11)	0.0438 (4)	
N3	0.19390 (8)	0.09876 (9)	0.25834 (12)	0.0464 (4)	
O1	0.10984 (12)	0.07594 (11)	−0.11088 (14)	0.1121 (7)	

### Atomic displacement parameters (Å^2^)

**Table:**

	*U*^11^	*U*^22^	*U*^33^	*U*^12^	*U*^13^	*U*^23^
C1	0.0422 (9)	0.0379 (10)	0.0420 (10)	0.0049 (8)	0.0027 (8)	0.0014 (8)
C2	0.0513 (11)	0.0412 (10)	0.0555 (12)	−0.0061 (9)	0.0021 (9)	−0.0001 (9)
C3	0.0583 (12)	0.0485 (11)	0.0555 (12)	−0.0063 (10)	−0.0070 (9)	−0.0080 (10)
C4	0.0599 (11)	0.0518 (12)	0.0402 (11)	−0.0027 (10)	−0.0033 (9)	−0.0020 (9)
C5	0.0404 (9)	0.0405 (10)	0.0415 (10)	0.0049 (8)	0.0026 (8)	0.0004 (8)
C6	0.0656 (12)	0.0715 (14)	0.0415 (11)	−0.0075 (11)	0.0011 (9)	0.0080 (10)
C7	0.0693 (13)	0.0733 (15)	0.0548 (13)	−0.0130 (12)	0.0074 (10)	0.0201 (11)
C8	0.0455 (10)	0.0474 (11)	0.0600 (12)	0.0034 (9)	0.0045 (9)	0.0108 (10)
C9	0.0523 (12)	0.0494 (13)	0.0841 (16)	−0.0028 (10)	0.0022 (11)	0.0159 (11)
C10	0.0525 (12)	0.0461 (13)	0.0930 (18)	−0.0048 (10)	−0.0075 (12)	0.0009 (12)
C11	0.0575 (12)	0.0569 (13)	0.0650 (13)	−0.0005 (11)	−0.0078 (10)	−0.0025 (11)
C12	0.0501 (10)	0.0461 (11)	0.0537 (12)	−0.0003 (9)	0.0010 (9)	0.0014 (9)
C13	0.0378 (9)	0.0380 (10)	0.0493 (11)	0.0060 (8)	0.0033 (8)	0.0006 (9)
C14	0.0634 (12)	0.0436 (11)	0.0515 (12)	−0.0067 (10)	−0.0049 (10)	0.0035 (9)
C15	0.0851 (15)	0.0409 (11)	0.0476 (12)	−0.0030 (11)	−0.0051 (11)	0.0012 (9)
C16	0.0813 (14)	0.0307 (10)	0.0469 (12)	0.0023 (10)	0.0109 (10)	0.0005 (9)
C17	0.117 (2)	0.0508 (13)	0.0559 (14)	0.0038 (14)	0.0237 (14)	−0.0006 (10)
C18	0.100 (2)	0.0672 (16)	0.088 (2)	−0.0025 (15)	0.0469 (16)	0.0001 (14)
C19	0.0740 (15)	0.0641 (15)	0.104 (2)	−0.0029 (12)	0.0309 (15)	−0.0014 (14)
C20	0.0591 (12)	0.0508 (12)	0.0729 (14)	0.0022 (10)	0.0118 (11)	0.0007 (10)
C21	0.0614 (12)	0.0323 (10)	0.0530 (12)	−0.0003 (9)	0.0097 (10)	0.0017 (9)
C22	0.125 (2)	0.0581 (14)	0.0584 (15)	−0.0025 (14)	−0.0222 (14)	−0.0043 (12)
N1	0.0427 (8)	0.0375 (8)	0.0422 (9)	0.0005 (7)	0.0031 (6)	0.0023 (7)
N2	0.0475 (8)	0.0438 (9)	0.0400 (9)	−0.0009 (7)	0.0039 (7)	0.0025 (7)
N3	0.0538 (9)	0.0405 (9)	0.0448 (9)	−0.0051 (7)	0.0049 (7)	0.0043 (7)
O1	0.189 (2)	0.0900 (13)	0.0577 (12)	0.0140 (13)	−0.0299 (12)	−0.0115 (9)

### Geometric parameters (Å, º)

**Table:**

C1—N1	1.331 (2)	C11—H11	0.9300
C1—C2	1.369 (2)	C12—C13	1.388 (2)
C1—N3	1.429 (2)	C12—H12	0.9300
C2—C3	1.375 (2)	C13—N2	1.400 (2)
C2—H2	0.9300	C14—C15	1.364 (2)
C3—C4	1.372 (2)	C14—N3	1.371 (2)
C3—H3	0.9300	C14—H14	0.9300
C4—C5	1.394 (2)	C15—C22	1.425 (3)
C4—H4	0.9300	C15—C16	1.432 (3)
C5—N1	1.3269 (19)	C16—C21	1.398 (2)
C5—N2	1.406 (2)	C16—C17	1.405 (3)
C6—C7	1.339 (3)	C17—C18	1.361 (3)
C6—N2	1.386 (2)	C17—H17	0.9300
C6—H6	0.9300	C18—C19	1.383 (3)
C7—C8	1.419 (3)	C18—H18	0.9300
C7—H7	0.9300	C19—C20	1.390 (3)
C8—C9	1.396 (3)	C19—H19	0.9300
C8—C13	1.405 (2)	C20—C21	1.372 (3)
C9—C10	1.362 (3)	C20—H20	0.9300
C9—H9	0.9300	C21—N3	1.408 (2)
C10—C11	1.386 (3)	C22—O1	1.215 (3)
C10—H10	0.9300	C22—H22	0.9300
C11—C12	1.384 (3)		
			
N1—C1—C2	124.85 (16)	C12—C13—C8	121.02 (17)
N1—C1—N3	113.26 (15)	N2—C13—C8	107.35 (16)
C2—C1—N3	121.88 (16)	C15—C14—N3	110.69 (17)
C1—C2—C3	116.90 (17)	C15—C14—H14	124.7
C1—C2—H2	121.5	N3—C14—H14	124.7
C3—C2—H2	121.5	C14—C15—C22	123.6 (2)
C4—C3—C2	120.36 (17)	C14—C15—C16	106.58 (17)
C4—C3—H3	119.8	C22—C15—C16	129.9 (2)
C2—C3—H3	119.8	C21—C16—C17	118.7 (2)
C3—C4—C5	117.99 (17)	C21—C16—C15	107.70 (16)
C3—C4—H4	121.0	C17—C16—C15	133.6 (2)
C5—C4—H4	121.0	C18—C17—C16	118.6 (2)
N1—C5—C4	122.65 (16)	C18—C17—H17	120.7
N1—C5—N2	116.02 (15)	C16—C17—H17	120.7
C4—C5—N2	121.32 (16)	C17—C18—C19	121.7 (2)
C7—C6—N2	110.53 (17)	C17—C18—H18	119.1
C7—C6—H6	124.7	C19—C18—H18	119.1
N2—C6—H6	124.7	C18—C19—C20	121.2 (2)
C6—C7—C8	107.79 (17)	C18—C19—H19	119.4
C6—C7—H7	126.1	C20—C19—H19	119.4
C8—C7—H7	126.1	C21—C20—C19	116.9 (2)
C9—C8—C13	119.42 (18)	C21—C20—H20	121.6
C9—C8—C7	133.48 (18)	C19—C20—H20	121.6
C13—C8—C7	107.10 (17)	C20—C21—C16	122.93 (17)
C10—C9—C8	119.50 (19)	C20—C21—N3	129.86 (18)
C10—C9—H9	120.2	C16—C21—N3	107.17 (16)
C8—C9—H9	120.2	O1—C22—C15	125.7 (3)
C9—C10—C11	120.73 (19)	O1—C22—H22	117.1
C9—C10—H10	119.6	C15—C22—H22	117.1
C11—C10—H10	119.6	C5—N1—C1	117.23 (14)
C12—C11—C10	121.54 (19)	C6—N2—C13	107.22 (14)
C12—C11—H11	119.2	C6—N2—C5	124.40 (15)
C10—C11—H11	119.2	C13—N2—C5	128.38 (14)
C11—C12—C13	117.77 (17)	C14—N3—C21	107.86 (15)
C11—C12—H12	121.1	C14—N3—C1	123.68 (14)
C13—C12—H12	121.1	C21—N3—C1	128.43 (15)
C12—C13—N2	131.59 (16)		
			
N1—C1—C2—C3	0.3 (3)	C19—C20—C21—N3	179.32 (18)
N3—C1—C2—C3	179.43 (15)	C17—C16—C21—C20	−1.1 (3)
C1—C2—C3—C4	−1.1 (3)	C15—C16—C21—C20	178.44 (17)
C2—C3—C4—C5	0.6 (3)	C17—C16—C21—N3	−179.34 (16)
C3—C4—C5—N1	0.6 (2)	C15—C16—C21—N3	0.19 (19)
C3—C4—C5—N2	179.67 (16)	C14—C15—C22—O1	179.0 (2)
N2—C6—C7—C8	−0.6 (2)	C16—C15—C22—O1	−0.8 (4)
C6—C7—C8—C9	−178.4 (2)	C4—C5—N1—C1	−1.4 (2)
C6—C7—C8—C13	0.5 (2)	N2—C5—N1—C1	179.54 (13)
C13—C8—C9—C10	0.2 (3)	C2—C1—N1—C5	0.9 (2)
C7—C8—C9—C10	178.9 (2)	N3—C1—N1—C5	−178.30 (13)
C8—C9—C10—C11	−1.0 (3)	C7—C6—N2—C13	0.6 (2)
C9—C10—C11—C12	0.6 (3)	C7—C6—N2—C5	−179.48 (16)
C10—C11—C12—C13	0.6 (3)	C12—C13—N2—C6	177.36 (18)
C11—C12—C13—N2	−178.68 (16)	C8—C13—N2—C6	−0.26 (18)
C11—C12—C13—C8	−1.3 (3)	C12—C13—N2—C5	−2.6 (3)
C9—C8—C13—C12	1.0 (3)	C8—C13—N2—C5	179.79 (15)
C7—C8—C13—C12	−178.03 (16)	N1—C5—N2—C6	169.29 (16)
C9—C8—C13—N2	178.90 (15)	C4—C5—N2—C6	−9.8 (2)
C7—C8—C13—N2	−0.11 (19)	N1—C5—N2—C13	−10.8 (2)
N3—C14—C15—C22	−179.03 (18)	C4—C5—N2—C13	170.11 (16)
N3—C14—C15—C16	0.8 (2)	C15—C14—N3—C21	−0.7 (2)
C14—C15—C16—C21	−0.6 (2)	C15—C14—N3—C1	−178.77 (16)
C22—C15—C16—C21	179.2 (2)	C20—C21—N3—C14	−177.78 (18)
C14—C15—C16—C17	178.8 (2)	C16—C21—N3—C14	0.30 (19)
C22—C15—C16—C17	−1.4 (4)	C20—C21—N3—C1	0.2 (3)
C21—C16—C17—C18	0.0 (3)	C16—C21—N3—C1	178.24 (16)
C15—C16—C17—C18	−179.4 (2)	N1—C1—N3—C14	34.4 (2)
C16—C17—C18—C19	0.7 (3)	C2—C1—N3—C14	−144.83 (17)
C17—C18—C19—C20	−0.3 (4)	N1—C1—N3—C21	−143.23 (16)
C18—C19—C20—C21	−0.8 (3)	C2—C1—N3—C21	37.5 (3)
C19—C20—C21—C16	1.5 (3)		

### Hydrogen-bond geometry (Å, º)

*Cg*5 is the centroid of the C16–C21 ring.

**Table:**

*D*—H···*A*	*D*—H	H···*A*	*D*···*A*	*D*—H···*A*
C6—H6···O1^i^	0.93	2.50	3.227 (3)	135
C12—H12···N1	0.93	2.41	2.931 (2)	116
C2—H2···*Cg*5^ii^	0.93	2.73	3.554 (2)	148

Symmetry codes: (i) *x*, *y*, *z*+1; (ii) *x*, −*y*+1/2, −*z*+1/2.
